# Inflammatory-neurological deficit versus metabolic dysregulation: unsupervised clustering and SHAP analysis

**DOI:** 10.3389/fimmu.2026.1879446

**Published:** 2026-07-15

**Authors:** Ziying Wang, Lingling Wang

**Affiliations:** 1 Department of Postgraduate, School of Clinical Medicine, Beihua University, Jilin, China; 2 Stroke Unit, Affiliated Hospital of Beihua University, Jilin, China

**Keywords:** C-reactive protein, inflammation, ischemic stroke, middle-aged and elderly, national institutes of health stroke scale

## Abstract

**Objective:**

This retrospective cohort study enrolled 20,538 middle-aged and older patients with acute ischemic stroke (AIS) to identify immune-metabolic clinical subtypes by unsupervised clustering, to examine the differential association between subtype characteristics and post-stroke epilepsy (PSE) susceptibility, and to clarify immune-related threshold biomarkers for individualized PSE risk stratification.

**Methods:**

Seven standardized variables were used for K-means clustering: age; National Institutes of Health Stroke Scale (NIHSS) score; lipid profile components (triglycerides, low-density lipoprotein cholesterol [LDL-c], high-density lipoprotein cholesterol [HDL-c]); glycated hemoglobin (HbA1c); and the immune-inflammatory marker C-reactive protein (CRP). Two immune-metabolic subtypes were determined. Multivariable logistic regression was performed to quantify the association between Cluster and PSE. An Extra Trees–based SHapley Additive exPlanations (SHAP) framework was used to interpret the hierarchical contribution of immune and metabolic indicators to PSE risk within each subtype.

**Results:**

Clustering produced two clinically distinct subtypes. Cluster 1, the inflammatory-neurological deficit cluster (n = 7,790), had older age, higher NIHSS scores, higher CRP, and higher HDL-c. Cluster 2, the metabolic dysregulation cluster (n = 12,748), had higher HbA1c, LDL-c, and triglycerides (TG). The incidence of PSE was substantially higher in the inflammatory-neurological deficit cluster than in the metabolic dysregulation cluster (7.2% vs. 2.4%, *P* < 0.001). After full adjustment, membership in the inflammatory-neurological deficit cluster was associated with 4.31-fold higher odds of PSE. Extra Trees–based SHAP analysis identified distinct drivers in each cluster. In the inflammatory-neurological deficit cluster, CRP emerged as the leading predictor with a risk-acceleration threshold above 24 mg/L. In the metabolic dysregulation cluster, NIHSS was the top predictor, and HbA1c values below 7% were associated with a protective effect.

**Conclusion:**

Unsupervised clustering separated AIS patients into two subtypes. The inflammatory-neurological deficit cluster had higher PSE risk. Cluster-specific SHAP results suggested candidate thresholds for individualized risk assessment.

## Introduction

Acute ischemic stroke (AIS) is a leading cause of death and long-term disability worldwide. In 2020, the global prevalence of all stroke subtypes reached 89.13 million, of which 68.16 million were AIS. Incident strokes totaled 11.71 million and AIS accounted for about 65% of new cases (1). Post-stroke epilepsy (PSE) is a serious complication of ischemic stroke. Reported cumulative incidence after AIS ranges from about 6.4% to 15% (2).

AIS is not only a focal brain injury but also a manifestation of systemic metabolic imbalance (3, 4). Core components of metabolic syndrome, including hyperglycemia and dyslipidemia, can significantly impair neurological recovery after stroke (5, 6). Systemic inflammatory markers contribute to metabolic disease progression and may promote epileptogenesis by disrupting the blood–brain barrier (7). Age and stroke severity are well-established predictors of outcome after AIS (2, 8). These risk factors often co-occur in patients with AIS. However, it remains unclear how they interact to cause PSE across different metabolic phenotypes, and there is a lack of large-sample studies that systematically stratify these phenotypes. Cluster analysis provides an alternative approach by grouping patients based on multiple variables to reveal potential synergistic patterns. Specifically, K-means clustering is one of the most robust and widely used methods within machine learning for data modeling (9–13). However, the interpretability of clustering results—the so-called “black box” problem—remains a major barrier to applying precision medicine (14). SHapley Additive exPlanations (SHAP) uses a game-theoretic framework to quantify feature contributions to model outputs, thereby improving the interpretability of cluster analyses (15).

In this cohort study of patients aged 45 years or older with AIS, we performed clustering using the following variables: age, National Institutes of Health Stroke Scale (NIHSS), triglycerides (TG), low-density lipoprotein cholesterol (LDL-c), high-density lipoprotein cholesterol (HDL-c), glycated hemoglobin (HbA1c), and C-reactive protein (CRP). We selected the 45-year threshold because stroke incidence increases markedly after this age (8). Multivariable regression was applied to evaluate the predictive value of clusters for PSE. Mediation analysis and subgroup analyses were explored. Finally, cluster-specific SHAP analyses were used to quantify each variable’s contribution, to enhance interpretability, and to support individualized assessment of PSE risk and prevention.

## Methods

### Study design and participants

**Figure 1:**
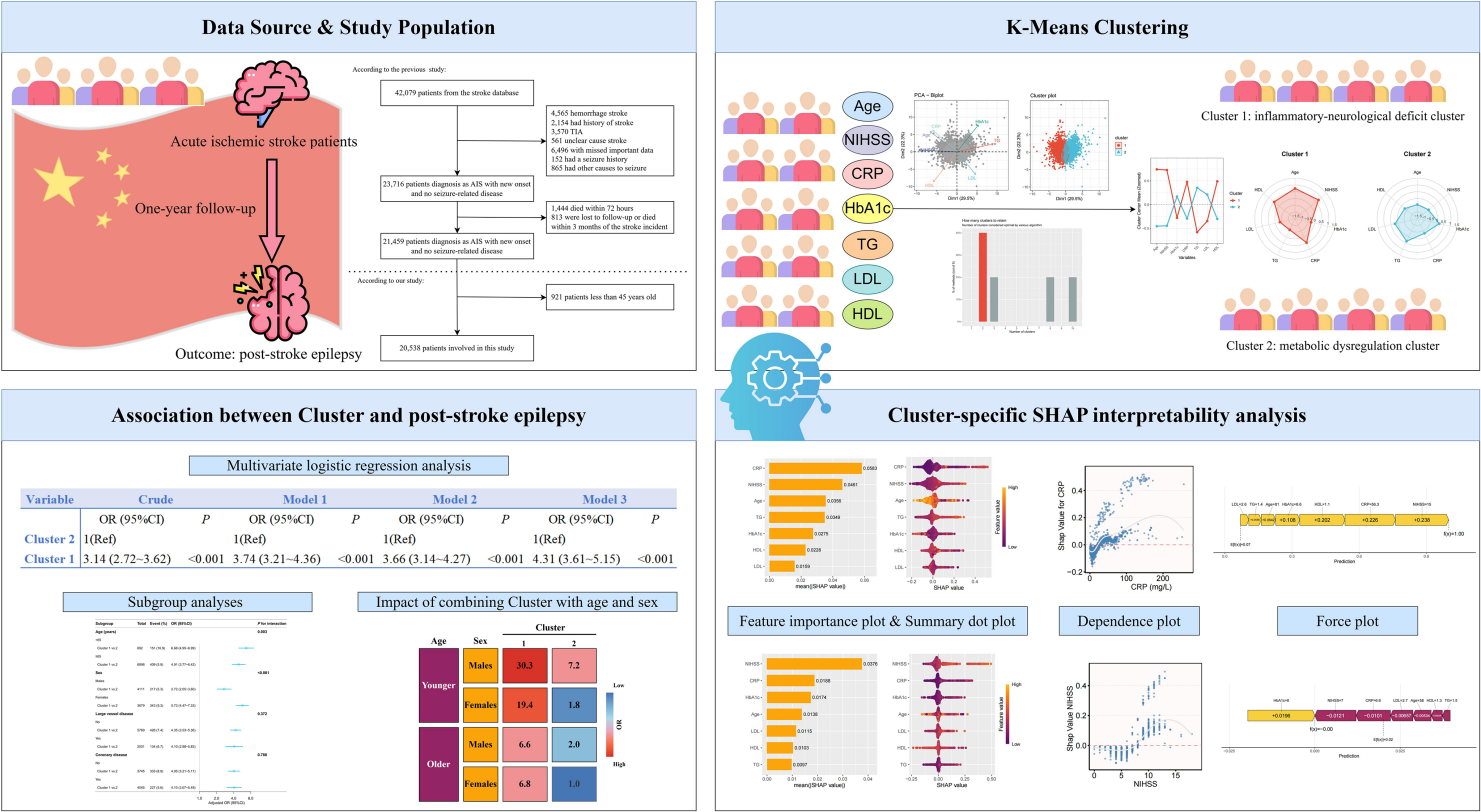
Overall flowchart of the study.

The retrospective study initially enrolled all stroke patients admitted to the Chongqing Emergency Center between June 2017 and June 2022 (https://datadryad.org/dataset/doi:10.5061/dryad.w0vt4b92c) (16). The protocol was approved by the Ethics Committee of Chongqing University Center Hospital. Eligible patients met two inclusion criteria: (1) age 18–90 years at admission and (2) a diagnosis of AIS that required hospitalization. Exclusion criteria were: (1) a prior stroke or transient ischemic attack (TIA); (2) conditions known to independently cause epilepsy (for example, traumatic brain injury, intracranial tumors, or cerebral vascular malformations); (3) a history of epilepsy or prior use of antiseizure medications for seizure prevention or for other indications (e.g., migraine or psychiatric disorders); (4) death within 72 hours of stroke onset; and (5) loss to follow-up (no outpatient records or unable to be contacted by telephone) or death within three months after stroke onset. For the present analysis, we limited the study population to adults aged 45 years or older to focus on the middle-aged and older group in which AIS incidence peaks (**Figure 1**) (8).

### Data collection

The following data were systematically collected at hospital admission for this study: clinical, imaging, and laboratory information. All variables were entered into the electronic medical record database within 24 hours of admission by the on-call neurologist or trained research staff, and were subsequently extracted and quality-checked centrally by the study team. Extracted items comprised demographic and clinical features (age, sex, NIHSS score on admission); past medical history (diabetes, hypertension, coronary disease, atrial fibrillation); complications (hydrocephalus); imaging information (cortical involvement, large vessel disease); and laboratory parameters (TG, LDL-c, HDL-c, HbA1c, CRP, estimated glomerular filtration rate [eGFR], alanine aminotransferase, urea, blood uric acid, platelet count, red blood cell count, activated partial thromboplastin time [APTT], D-dimer). The definitions and measurement methods were as follows. NIHSS score was assessed and recorded on admission by neurologists who had received standardized training. Diabetes, hypertension, and coronary disease were identified from prior inpatient or outpatient records. Atrial fibrillation was defined by a prior diagnosis or by atrial fibrillation/atrial flutter on the admission electrocardiogram. Hydrocephalus was defined by imaging evidence of ventricular enlargement with corresponding clinical signs. Large vessel disease was determined according to TOAST classification principles combined with vascular imaging (CTA, MRA, or DSA). “Cortical involvement” was recorded if the infarct affected any of the frontal, parietal, temporal, or occipital lobes, or the insula.

AIS constitutes an acute exacerbation of chronic systemic processes (17). NIHSS and CRP capture the acute pathophysiological state at admission, while HbA1c and the lipid profile indicate chronic metabolic traits. By integrating these dimensions at the critical time of stroke onset, we aim to capture the convergence of acute injury severity and underlying metabolic susceptibility. All blood samples were collected within 24 hours of admission, and laboratory parameters were obtained from the first measurements performed at admission. These first tests most closely represent the baseline inflammatory and metabolic status prior to fluid resuscitation or pharmacological therapy, thereby minimizing the influence of early clinical interventions on the laboratory results. Lipids (TG, LDL-c, HDL-c) were measured by isotopic methods or routine enzymatic colorimetry. HbA1c was measured by high−performance liquid chromatography. CRP was measured by immunoturbidimetry or high-sensitivity CRP assays. Creatinine was measured by an IDMS-traceable enzymatic method or by the Jaffe method. Alanine aminotransferase, urea, blood uric acid, and other biochemical parameters were measured on the hospital central laboratory’s automated biochemistry analyzers, and blood cell counts were performed on automated hematology analyzers. APTT and D-dimer were measured on automated coagulation analyzers according to the manufacturers’ instructions. eGFR was calculated using the revised Modification of Diet in Renal Disease equation with adjustment for the Chinese population and is reported in mL/min/1.73 m^2^ (18).

### Outcome

The primary outcome was PSE occurring within one year after AIS. PSE was defined as two or more unprovoked seizures occurring after the acute phase of stroke. Under the updated International League Against Epilepsy criteria, a single unprovoked seizure with a high risk of recurrence was also considered PSE (19). PSE was identified during follow-up and confirmed through outpatient re-evaluations and telephone interviews conducted by neurologists. Patients were excluded from the PSE endpoint analysis if they were lost to follow-up (no outpatient records or unable to be contacted by telephone) or if they died within three months of stroke onset.

### Statistical analysis

Indicators with more than 10% missing data were excluded. Remaining indicators with missing values were imputed using the random forest algorithm with default parameters. Features were processed in order of increasing missingness to minimize imputation complexity. During imputation, missing values in other features were temporarily replaced with 0, and predicted values were inserted into the original feature matrix before moving to the next feature. This process continued until all features were complete.

Prior to clustering, the seven clustering variables (age, NIHSS, TG, LDL-c, HDL-c, HbA1c, and CRP) were standardized using Z-scores to remove scale differences. Principal component analysis (PCA) was used to reduce the dimensionality of the dataset. After dimensionality reduction using PCA, K-means clustering was applied to group patients based on the similarity of their feature profiles. The optimal cluster number was identified using a consensus strategy integrating results from easystats, NbClust, and mclust. Internal validity indices (Silhouette, Calinski–Harabasz, Davies–Bouldin, and Gap statistics), majority-rule criteria from NbClust, and Bayesian Information Criterion (BIC)-based model selection from Gaussian mixture models were jointly considered, and the cluster solution supported by the majority of methods was retained. Clinical interpretability was also taken into account when selecting the final solution.

Continuous variables with approximate normal distributions are reported as mean ± standard deviation (SD). Skewed continuous variables are reported as median and interquartile range (IQR). Categorical variables are presented as counts and percentages (%). Between-group or between-cluster comparisons of continuous variables used the independent samples Student’s t-test or the Mann–Whitney U-test as appropriate. Categorical variables were compared using the chi-square test.

Multivariable logistic regression analyses were performed and odds ratios (ORs) with 95% confidence intervals (CIs) were reported. Covariates included in the multivariable models were selected based on prior literature and clinical relevance (8, 16, 20–28). To evaluate robustness, we specified three hierarchical adjustment models. Model 1 was adjusted for sex, diabetes, hypertension, coronary disease, atrial fibrillation, and hydrocephalus. Model 2 included the covariates in Model 1 and additionally adjusted for cortical involvement and large vessel disease. Model 3 further adjusted for eGFR, alanine aminotransferase, urea, blood uric acid, platelet count, red blood cell count, and APTT.

Subgroup effects were examined using likelihood ratio tests. After observing interactions between Cluster and sex, and between Cluster and age, we cross-classified participants by age, sex, and Cluster. Then a multivariable logistic regression model was fitted to estimate ORs for each resulting stratum. A mediation analysis was conducted using bootstrap resampling (1,000 iterations) to estimate the mediating effect of D-dimer on the association between Cluster and PSE.

For each cluster, the input features comprised age, NIHSS, TG, LDL-c, HDL-c, HbA1c, and CRP. Four diverse machine learning algorithms were implemented to provide a comprehensive comparative analysis in each cluster: Extra Trees (ET), Light Gradient Boosting Machine (LightGBM), Random Forest (RF), and CatBoost. Model performance was assessed using a multi-metric framework. Sensitivity, specificity, F1 score (the harmonic mean of precision and recall), and overall accuracy were calculated to assess classification performance across different aspects. The area under the receiver operating characteristic curve (AUROC) evaluated the models’ ability to distinguish between positive and negative cases across various decision thresholds. The area under the precision-recall curve (AUPRC) specifically focused on positive-case identification, which is particularly important for imbalanced datasets. PSE constitutes a minority-class outcome. To address class imbalance, we used the Synthetic Minority Oversampling Technique (SMOTE), which generates synthetic minority-class samples rather than duplicating existing records. SMOTE generally outperforms simple oversampling and is widely used for imbalanced datasets (29). A dual validation strategy (stratified 5-fold cross-validation on the training set and evaluation on an independent test set) was used to mitigate overfitting.

To quantify the contribution of each input feature to PSE risk prediction within each cluster, Kernel SHAP was applied to compute SHAP values. Three complementary visualization methods were used: (1) SHAP feature importance and summary dot plots to show the distribution and relative importance of features across the training set; (2) partial dependence plots using locally weighted scatterplot smoothing (LOWESS) to explore potential non-linear relationships between individual features and their SHAP values; and (3) SHAP force plots to decompose individual-level predictions and visually demonstrate how each feature shifted the prediction.

Two sensitivity analyses were performed to evaluate the robustness of our clustering results. First, to reduce the influence of extreme values, we winsorized NIHSS, CRP, and TG at the 99th percentile and repeated K-means clustering. Second, to address distributional skewness, we applied natural log transformations to NIHSS, CRP, and TG and repeated K-means clustering. In both analyses, the number of clusters was fixed *a priori* at two. Concordance between the primary clustering and each sensitivity-derived solution was assessed using Cohen’s kappa; we reran the multivariable logistic regression models to verify whether the associations remained consistent.

Analysis was performed using R 4.2.2 (http://www.Rproject.org; The R Foundation, Vienna, Austria) and the Free Statistics software (version 2.5; Beijing FreeClinical Medical Technology Co., Ltd, Beijing, China). A two-tailed *P*-value < 0.05 was considered statistically significant.

## Results

### Basic characteristics of the population

**Supplementary Table 1** summarizes the clinical characteristics of the cohort stratified by PSE status. At one year, among 20,538 middle-aged and older AIS patients, 867 developed PSE. Compared with patients who did not develop PSE, PSE cases were more often female, had higher stroke severity as measured by NIHSS, and had higher TG, CRP, and D-dimer levels (*P* < 0.001).

### Clinical characteristics of the two clusters

The consensus strategy shows that the optimal cluster number was 2 (**Supplementary Figure 1**). Furthermore, solutions with three or more clusters produced subgroups with sparse outcome events, reducing their clinical usefulness. We therefore selected a two-cluster solution (k = 2) as optimal by balancing the consensus strategy with practical clinical considerations.

**Figure 2:**
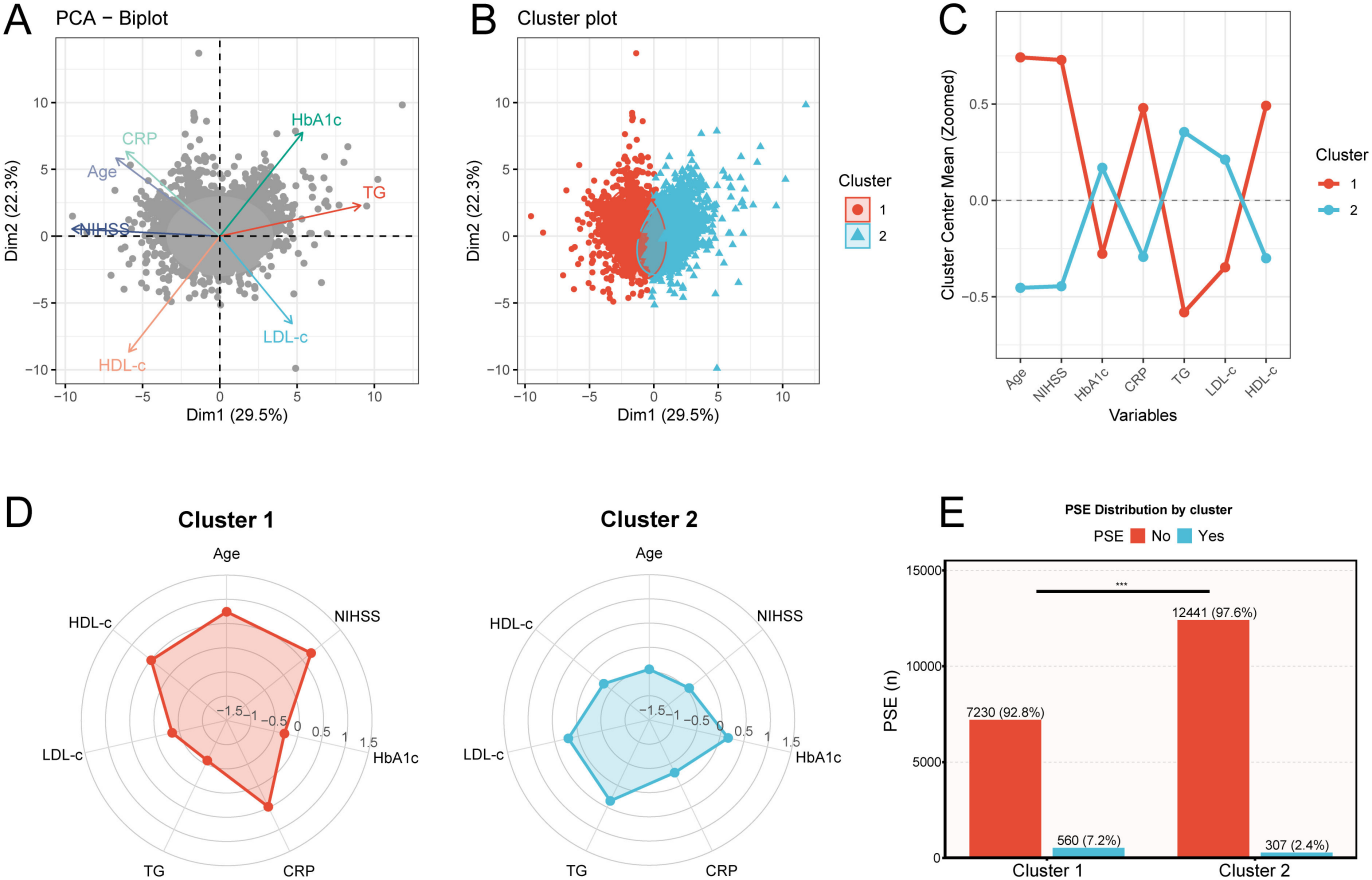
Identification of subtypes in middle-aged and older patients with AIS. PCA biplot **(A)**; K-means clustering results (k = 2) **(B)**; Cluster profiles **(C, D)**; Cluster 1, inflammatory-neurological deficit cluster, showed older age, higher CRP, higher HDL-c, and higher NIHSS; Cluster 2, metabolic dysregulation cluster, showed higher HbA1c, higher LDL-c, and higher TG; PSE distribution by Cluster **(E)**. AIS, acute ischemic stroke; PCA, Principal Component Analysis; CRP, C-reactive protein; HDL-c, high-density lipoprotein cholesterol; NIHSS, National Institutes of Health Stroke Scale; HbA1c, glycated hemoglobin; LDL-c, low-density lipoprotein cholesterol; TG, triglycerides; PSE, post-stroke epilepsy.

As shown in **Figure 2**, Cluster 1 had older age, higher NIHSS, higher CRP, and higher HDL-c. Cluster 2 had higher HbA1c, LDL-c, and TG. Cluster 1 was characterized as the inflammatory-neurological deficit cluster. Cluster 2 was characterized as the metabolic dysregulation cluster.

**Table 1** presents clinical characteristics for the 20,538 participants by Cluster (inflammatory-neurological deficit cluster, n = 7,790; metabolic dysregulation cluster, n = 12,748). Except for large vessel disease (*P* = 0.12), all between-cluster differences were statistically significant (all *P* < 0.001). Compared with the metabolic dysregulation cluster, the inflammatory-neurological deficit cluster had higher proportions of males, coronary disease, atrial fibrillation, hydrocephalus, and cortical involvement, while the proportions with diabetes and hypertension were significantly lower. Biochemically, the inflammatory-neurological deficit cluster had lower eGFR, blood uric acid, platelet count, and red blood cell count, and higher alanine aminotransferase, urea, D-dimer, and APTT. The incidence of PSE was 7.2% in the inflammatory-neurological deficit cluster, significantly higher than 2.4% in the metabolic dysregulation cluster (*P* < 0.001, **Figure 2E**).

**Table 1:** Comparative analysis of relevant features between clusters.

Variables	Total	Cluster 1	Cluster 2	P
	(n = 20,538)	(n = 7,790)	(n = 12,748)	
Sex (Female), n (%)	10,260 (49.96)	3,679 (47.23)	6,581 (51.62)	< 0.001
Diabetes, n (%)	7,167 (34.90)	1,770 (22.72)	5,397 (42.34)	< 0.001
Hypertension, n (%)	14,308 (69.67)	4,789 (61.48)	9,519 (74.67)	< 0.001
Coronary disease, n (%)	9,521 (46.36)	4,045 (51.93)	5,476 (42.96)	< 0.001
Atrial fibrillation, n (%)	2,024 (9.85)	1,386 (17.79)	638 (5.00)	< 0.001
Hydrocephalus, n (%)	250 (1.22)	168 (2.16)	82 (0.64)	< 0.001
Cortical involvement, n (%)	1,255 (6.11)	658 (8.45)	597 (4.68)	< 0.001
Large vessel disease, n (%)	5,401 (26.30)	2,001 (25.69)	3,400 (26.67)	0.120
eGFR (mL/min/1.73m²), Mean ± SD	88.82 ± 29.20	84.08 ± 28.29	91.72 ± 29.37	< 0.001
Alanine aminotransferase (U/L), Mean ± SD	24.28 ± 10.29	24.71 ± 10.57	24.01 ± 10.11	< 0.001
Urea (mmol/L), Mean ± SD	6.47 ± 1.41	6.58 ± 1.21	6.40 ± 1.51	< 0.001
Blood uric acid (µmol/L), Mean ± SD	343.15 ± 58.62	328.35 ± 55.87	352.20 ± 58.42	< 0.001
Platelet count (109/L), Mean ± SD	189.62 ± 26.86	180.38 ± 23.16	195.26 ± 27.40	< 0.001
Red blood cell count (1012/L), Mean ± SD	4.31 ± 0.32	4.21 ± 0.30	4.36 ± 0.32	< 0.001
APTT (s), Mean ± SD	35.67 ± 2.32	36.54 ± 2.46	35.14 ± 2.06	< 0.001
D-dimer (µg/mL), Median (IQR)	0.91 (0.65, 1.50)	1.34 (0.87, 2.10)	0.77 (0.55, 1.11)	< 0.001

Cluster 1, inflammatory-neurological deficit cluster; Cluster 2, metabolic dysregulation cluster; eGFR, estimated glomerular filtration rate; SD, standard deviation; IQR, interquartile range; APTT, activated partial thromboplastin time; PSE, post-stroke epilepsy.

### Association between cluster and PSE

As shown in **Table 2**, in the unadjusted model, the inflammatory-neurological deficit cluster had 3.14-fold higher odds of PSE than the metabolic dysregulation cluster (OR 3.14, 95% CI 2.72–3.62, *P* < 0.001). This association remained significant in Model 3, with the inflammatory-neurological deficit cluster demonstrating 4.31-fold higher odds of PSE compared with the metabolic dysregulation cluster (OR 4.31, 95% CI 3.61–5.15, *P* < 0.001).

**Table 2:** Association between cluster and PSE.

Variable	Crude		Model 1		Model 2		Model 3	
	OR (95%CI)	P	OR (95%CI)	P	OR (95%CI)	P	OR (95%CI)	P
Metabolic dysregulation cluster	1 (Ref)		1 (Ref)		1 (Ref)		1 (Ref)	
Inflammatory-neurological deficit cluster	3.14 (2.72~3.62)	<0.001	3.74 (3.21~4.36)	<0.001	3.66 (3.14~4.27)	<0.001	4.31 (3.61~5.15)	<0.001

Model 1: Adjusted for sex, diabetes, hypertension, coronary disease, atrial fibrillation, and hydrocephalus.

Model 2: Adjusted for sex, diabetes, hypertension, coronary disease, atrial fibrillation, hydrocephalus, cortical involvement, and large vessel disease.

Model 3: Adjusted for sex, diabetes, hypertension, coronary disease, atrial fibrillation, hydrocephalus, cortical involvement, large vessel disease, eGFR, alanine aminotransferase, urea, blood uric acid, platelet count, red blood cell count, and APTT. OR, odds ratio; CI, confidence interval; eGFR, estimated glomerular filtration rate; APTT, activated partial thromboplastin time; PSE, post-stroke epilepsy.

### Subgroup and mediation analysis

**Figure 3:**
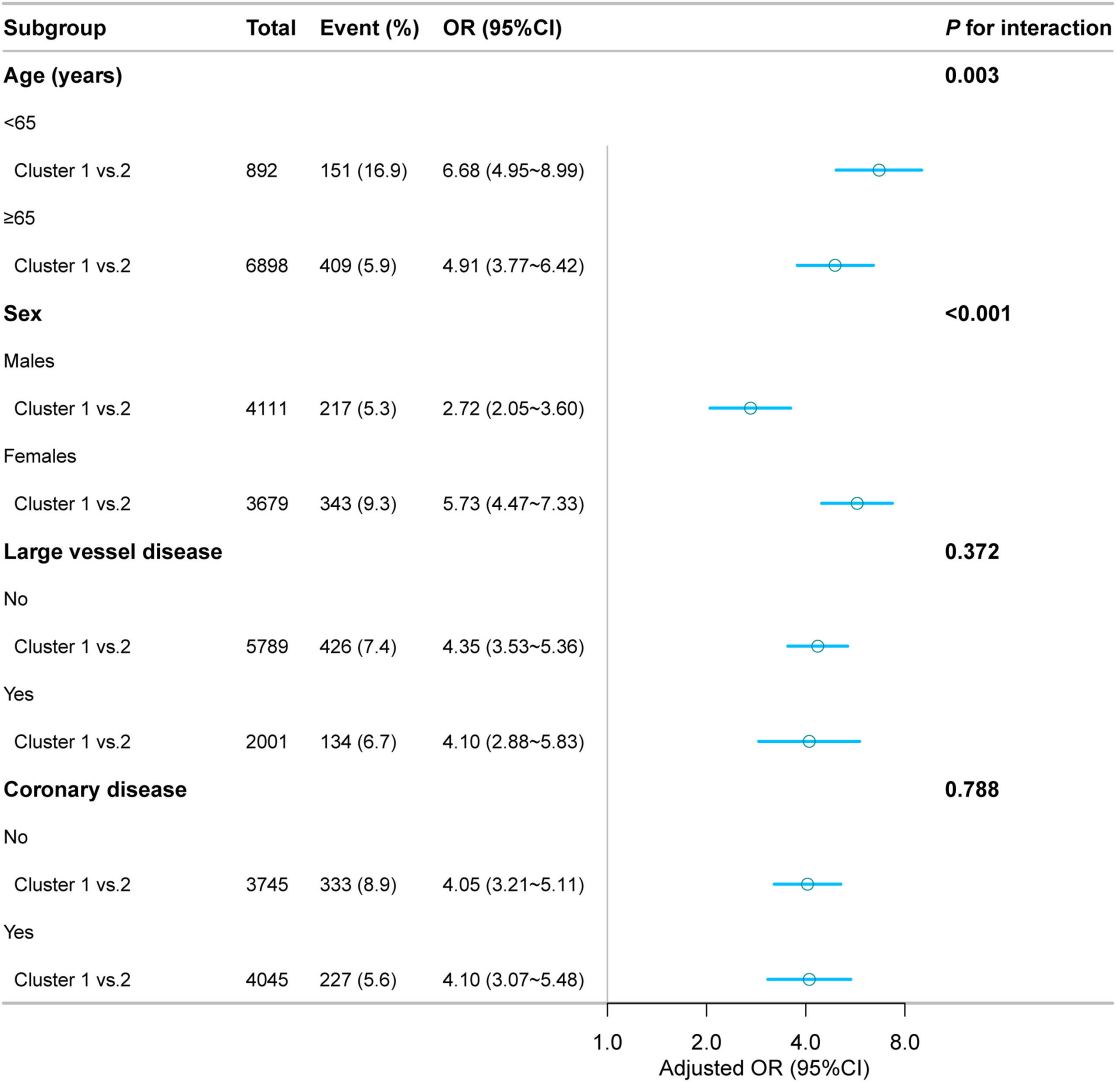
Subgroup analyses. Adjusted for sex, diabetes, hypertension, coronary disease, atrial fibrillation, hydrocephalus, cortical involvement, large vessel disease, eGFR, alanine aminotransferase, urea, blood uric acid, platelet count, red blood cell count, and APTT. For subgroup analyses conducted within levels of a categorical variable, that variable was not included as a covariate. Cluster 1, inflammatory-neurological deficit cluster; Cluster 2, metabolic dysregulation cluster; OR, odds ratio; CI, confidence interval; eGFR, estimated glomerular filtration rate; APTT, activated partial thromboplastin time; PSE, post-stroke epilepsy.

**Figure 4:**
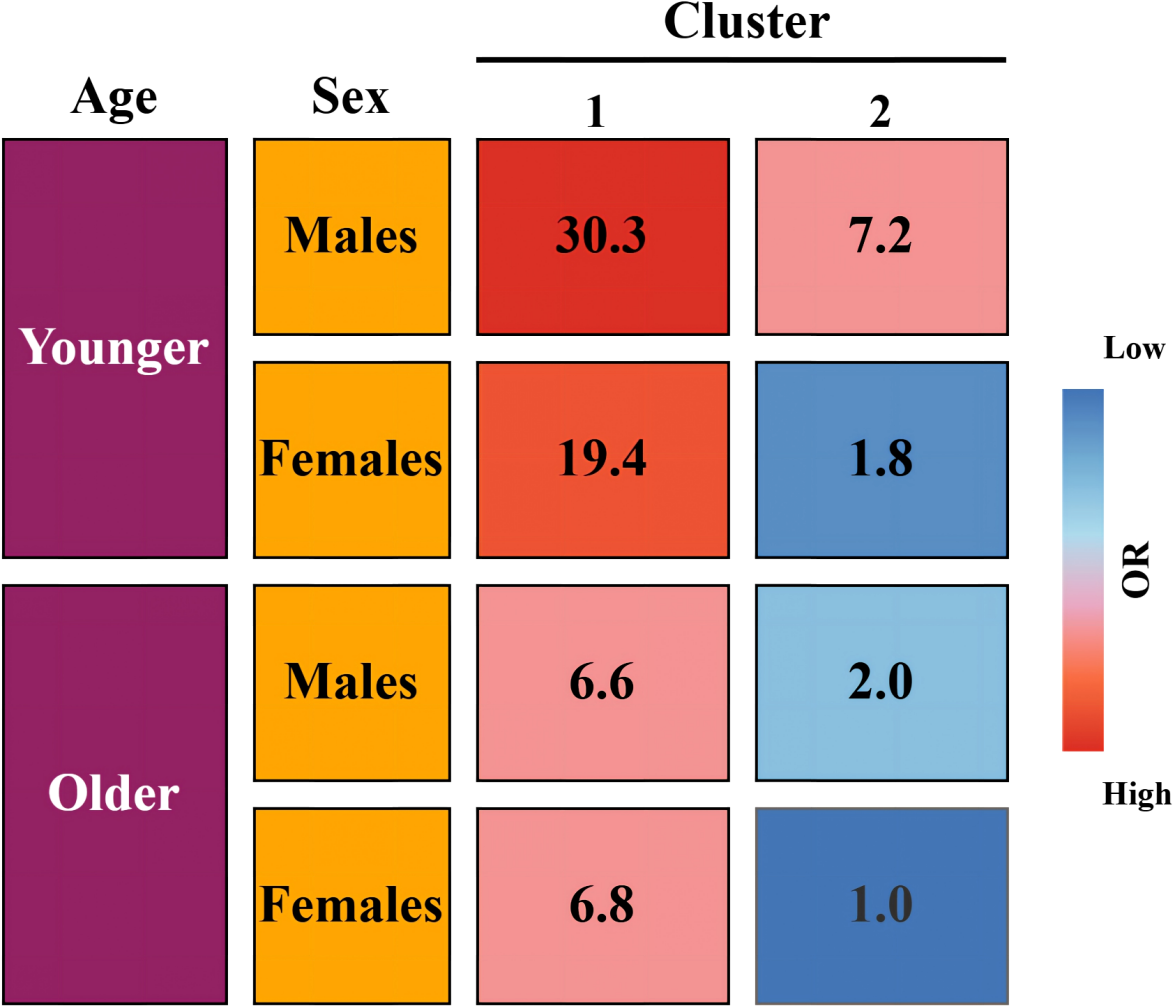
Impact of combining cluster with age and sex on the risk of PSE. Adjusted for diabetes, hypertension, coronary disease, atrial fibrillation, hydrocephalus, cortical involvement, large vessel disease, eGFR, alanine aminotransferase, urea, blood uric acid, platelet count, red blood cell count, and APTT. All *P* < 0.05. Cluster 1, inflammatory-neurological deficit cluster; Cluster 2, metabolic dysregulation cluster; Younger, 45–65 years old; Older, ≥65 years old; OR, odds ratio; eGFR, estimated glomerular filtration rate; APTT, activated partial thromboplastin time; PSE, post-stroke epilepsy.

Subgroup analyses revealed interactions between Cluster and sex and between Cluster and age (**Figure 3**). After cross-classification, ORs for PSE, from highest to lowest, were as follows: younger males with inflammatory-neurological deficits; younger females with inflammatory-neurological deficits; younger males with metabolic dysregulation; older females with inflammatory-neurological deficits; older males with inflammatory-neurological deficits; older males with metabolic dysregulation; younger females with metabolic dysregulation; and older females with metabolic dysregulation (**Figure 4**).

**Supplementary Figure 2** illustrates a potential mediating role of D-dimer in the association between Cluster and PSE. Compared with the metabolic dysregulation cluster, the inflammatory-neurological deficit cluster was associated with higher D-dimer levels (β = 0.63, 95% CI 0.59–0.68). Higher D-dimer was independently associated with increased PSE risk (OR = 2.13, 95% CI 2.02–2.25). Exploratory mediation analysis suggested that D-dimer may account for a proportion of the association between Cluster and PSE (mediated proportion 34.38%, 95% CI 28.88%–42.01%, *P* < 0.001).

### Cluster-specific SHAP interpretability analysis

Benchmarking evaluations on the training set across the four algorithms showed that the ET model achieved the highest AUROC in both clusters (metabolic dysregulation: 0.99; inflammatory-neurological deficit: 0.97), indicating superior discriminative performance relative to the other algorithms (**Supplementary Figures 3**, **4**). AUPRC values further supported the ET model’s clinical utility in both clusters. The ET model also demonstrated strong performance across multiple evaluation metrics, with consistent results between training and test evaluations (**Supplementary Figures 5**–**8**). Based on these overall performance assessments, the ET model was chosen for the final SHAP interpretability analysis.

**Figure 5:**
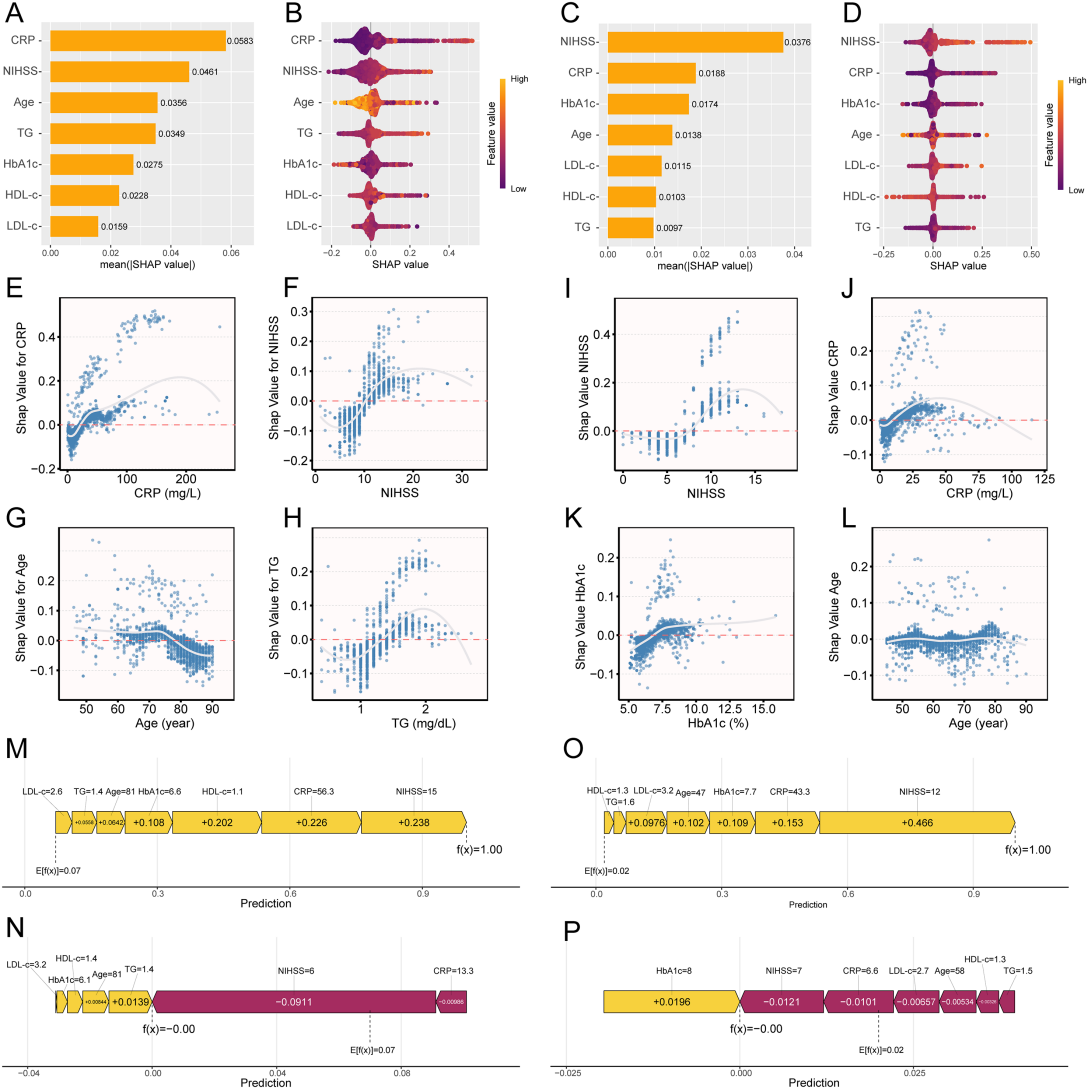
Cluster-specific SHAP interpretability analysis. SHAP feature importance plots in inflammatory-neurological deficit cluster **(A)** and metabolic dysregulation cluster **(C)**; SHAP summary dot plots in inflammatory-neurological deficit cluster **(B)** and metabolic dysregulation cluster **(D)**; SHAP dependence plots in inflammatory-neurological deficit cluster **(E–H)** and metabolic dysregulation cluster **(I–L)**; SHAP force plots for non-PSE in inflammatory-neurological deficit cluster **(M)**; SHAP force plots for PSE in inflammatory-neurological deficit cluster **(N)**; SHAP force plots for non-PSE in metabolic dysregulation cluster **(O)**; SHAP force plots for PSE in metabolic dysregulation cluster **(P)**. SHAP, SHapley Additive exPlanations; CRP, C-reactive protein; HDL-c, high-density lipoprotein cholesterol; NIHSS, National Institutes of Health Stroke Scale; HbA1c, glycated hemoglobin; LDL-c, low-density lipoprotein cholesterol; TG, triglycerides; PSE, post-stroke epilepsy.

SHAP analysis based on the ET model was used to evaluate the relative importance and directional effects of the seven variables for PSE prediction within each cluster. The model’s hyperparameters are listed in **Supplementary Table 2**. In the inflammatory-neurological deficit cluster, SHAP feature importance ranked CRP as the most influential variable, followed by NIHSS, age, and TG (**Figure 5A**). The SHAP summary dot plot (**Figure 5B**) showed that higher CRP, NIHSS, and TG values were associated with increased PSE risk. In the metabolic dysregulation cluster, NIHSS was the most important predictor, followed by CRP, HbA1c, and age (**Figure 5C**). The summary dot plot (**Figure 5D**) indicated that higher NIHSS, CRP, and HbA1c were associated with increased PSE risk.

The SHAP dependence plots revealed clinically relevant threshold effects. In the inflammatory-neurological deficit cluster (**Figures 5E–H**), CRP exhibited a positive relationship with PSE risk that accelerated above 24 mg/L. NIHSS demonstrated a threshold effect near 11. Age showed a protective effect above 79 years. TG conferred protection most substantially at values below 1.4 mmol/L. In the metabolic dysregulation cluster (**Figures 5I–L**), NIHSS demonstrated a threshold effect near 8. CRP exhibited a positive relationship with PSE risk that accelerated above 10.1 mg/L. HbA1c showed a protective effect at values below 7%.

SHAP force plots illustrated additive explanations for two contrasting individual predictions (**Figures 5M–P**). For example, in the metabolic dysregulation cluster, for a representative participant with a predicted PSE probability close to 0 (**Figure 5P**), the force plot shows how individual factors influenced that prediction: elevated HbA1c (8%) increased PSE risk, whereas lower NIHSS (7), lower CRP (6.6 mg/L), lower LDL-c (2.7 mmol/L), lower TG (1.5 mmol/L), and moderate age (58 years) and HDL-c (1.3 mmol/L) collectively exerted protective effects.

### Sensitivity analysis

The clustering solutions after winsorization and after log transformation showed high agreement with the primary clustering (Cohen’s kappa = 0.977 and 0.866, respectively) (**Supplementary Figures 9**, **10**). These kappa values indicate that cluster membership remained largely unchanged when outliers were down-weighted or when skewness was addressed. The multivariable regression results were broadly consistent across sensitivity analyses (**Supplementary Tables 3**, **4**), supporting the robustness of the associations identified in the primary analysis.

## Discussion

In this large cohort of middle-aged and older adults with AIS, unsupervised clustering identified two subtypes. The associations of these subtypes with PSE were investigated. Cluster 1, the inflammatory-neurological deficit cluster (n = 7,790) had older age, higher NIHSS, higher CRP, and higher HDL-c. Cluster 2, the metabolic dysregulation cluster, (n = 12,748) had higher HbA1c, LDL-c, and TG. After adjustment for covariates, patients in the inflammatory-neurological deficit cluster had 4.31 times the risk of PSE compared with patients in the metabolic dysregulation cluster. Subgroup analyses revealed interactions between Cluster and sex and between Cluster and age. After cross-stratifying by Cluster, sex, and age, OR for PSE was highest among younger males in the inflammatory-neurological deficit cluster. Exploratory mediation analysis suggested that D-dimer may partially mediate the association, with an estimated mediated proportion of 34.38%. Explainable machine learning models were validated within each cluster. The models showed that CRP and NIHSS were the most important predictors in the inflammatory-neurological deficit cluster and in the metabolic dysregulation cluster. SHAP analysis identified threshold values for these markers that may be clinically useful.

The inflammatory-neurological deficit cluster had older age, higher NIHSS, higher CRP, and higher HDL-c. The higher PSE risk in the inflammatory-neurological deficit cluster may arise from neuroinflammation combined with more severe ischemic injury. Elevated CRP indicated a strong systemic inflammatory response. This response can worsen blood-brain barrier disruption. It can also promote glutamate excitotoxicity and drive epileptogenesis through cytokine pathways (30). Higher NIHSS on admission indicated larger or more severe infarction. The risk of acute symptomatic seizures after ischemic stroke rises with higher NIHSS on admission (31). HDL-c is usually seen as protective. Its elevation in this cluster may reflect a compensatory anti-inflammatory response that is insufficient to counter the proinflammatory state. The metabolic dysregulation cluster showed higher HbA1c, LDL-c, and TG. These findings indicate metabolic dysregulation. Increased LDL-c and TG have been linked to epileptogenesis through mechanisms such as ferroptosis, autophagy and lysosomal dysfunction, and changes in the gut microbiota (32). Elevated HbA1c signaled poor recent glycemic control. Hyperglycemia can worsen ischemic injury by producing reactive oxygen species, promoting cerebral edema and reperfusion damage, and impairing mitochondrial function. These effects can increase infarct size and raise the risk of hemorrhagic transformation (20).

It has long been thought that females have a lower risk of developing epilepsy than males (21–23, 33, 34). However, among elderly patients in the inflammatory-neurological deficit cluster, advanced age and the age−related decline in estrogen levels may attenuate estrogen−mediated neuroprotection, thereby exacerbating inflammatory responses, disrupting blood–brain barrier homeostasis, and perturbing downstream processes of neuronal injury and repair (35), resulting in a higher risk of PSE in females than in males. In clinical practice, emphasis should be placed on heightened vigilance, early screening, and individualized interventions for high−risk groups. For stroke patients with a high overall inflammatory burden and severe neurological deficits, the risk of seizures should be closely monitored regardless of age or sex. For the metabolic dysregulation subtype, although the overall risk is relatively low, attention should still be paid to the potential risk in younger males. D-dimer emerged as a potential intermediate biomarker linking the inflammatory-neurological deficit cluster to PSE. This cluster, characterized by inflammation and a high injury phenotype, may contribute to PSE risk, possibly through coagulation activation reflected by D-dimer. Although preliminary, these exploratory findings raise the possibility that anticoagulant strategies could be investigated for PSE prevention in high-risk groups. Further interventional studies are needed before any clinical application.

ET model with SHAP explainability was used to rank predictors and to reveal threshold effects. In the inflammatory-neurological deficit cluster, CRP emerged as the most influential predictor. CRP surpassed even the traditional neurological deficit score, NIHSS. This result underscores the central role of systemic inflammation in PSE pathogenesis for this subtype. The effect of CRP on PSE risk was nonlinear. Risk accelerated when CRP exceeded 24 mg/L, providing a potential clinical high-risk alert threshold. Patients older than 79 years had reduced PSE risk in this cluster. This finding is consistent with reports that younger patients may have relatively higher PSE risk (36–38). By contrast, in the metabolic dysregulation cluster, NIHSS replaced CRP as the primary predictor. This indicates that PSE risk in this subtype is more dependent on the severity of initial brain injury. HbA1c was also substantially important in the metabolic dysregulation cluster. Values below 7% were protective, supporting the clinical value of glycemic control for preventing PSE in metabolically dysregulated patients. The CRP risk acceleration threshold in the metabolic dysregulation cluster was 10.1 mg/L. This threshold was much lower than the 24 mg/L threshold for the inflammatory-neurological deficit cluster, indicating that metabolically dysregulated patients have lower tolerance to inflammatory perturbations and that even modest CRP elevations can markedly increase PSE risk. These findings collectively emphasize the need for cluster-based individualized risk assessment and suggest that metabolic dysregulation may reduce the brain’s tolerance to inflammatory injury. For example, in a metabolically imbalanced environment, even mild systemic inflammation can trigger a pronounced pro-epileptogenic effect.

Nevertheless, several limitations merit consideration. First, the retrospective design restricted analyses to recorded variables. Important confounders such as medication use were unavailable and thus unadjusted. To assess potential unmeasured confounding, we computed the E-value, which was 8.09 overall and 6.68 for the lower confidence limit. Hence, the observed OR of 4.31 could be explained only by an unmeasured confounder linked to both Cluster and PSE with an OR ≥ 8.09 beyond measured covariates, whereas weaker confounding could not account for it. Second, biomarker measurements were confined to the first 24 hours post-admission, capturing initial inflammatory and metabolic states but not their longitudinal changes. D-dimer and the clustering variables were measured simultaneously, and therefore the mediation findings may decompose associations without proving temporal causality. Third, although we applied multiple optimization measures to address class imbalance, the SHAP analysis remains exploratory and requires prospective validation. Fourth, data from Dryad had prior imputation, precluding precise missingness reporting and complete-case sensitivity analyses, though original exclusions and random forest imputation likely reduced bias. Finally, excluding prior stroke or TIA limits generalizability to first-ever ischemic stroke, and broader cohorts are needed. Regional recruitment in southwest China may constrain applicability and necessitates replication in multiethnic populations.

## Conclusion

Unsupervised clustering separated AIS patients into two reproducible subtypes with different PSE risks. One subtype showed inflammatory features and severe neurological deficit and had high PSE risk. The other subtype showed metabolic dysregulation and had lower PSE risk. The findings highlight systemic inflammation and stroke severity as important factors for PSE. Cluster-specific SHAP analysis suggested thresholds may support individualized risk assessment and prevention.

## Data Availability

Publicly available datasets were analyzed in this study. This data can be found here: https://datadryad.org/dataset/doi:10.5061/dryad.w0vt4b92c.
