# Supplementary material for: Inflammatory-neurological deficit versus metabolic dysregulation: unsupervised clustering and SHAP analysis
Source: Front Immunol. 2026 Jul 15;17:1879446. doi: 10.3389/fimmu.2026.1879446 (PMC13414985; doi:10.3389/fimmu.2026.1879446)
Supplement: Supplementary file 1 [file DataSheet1.docx]

Supplementary Material

# Supplementary Figures


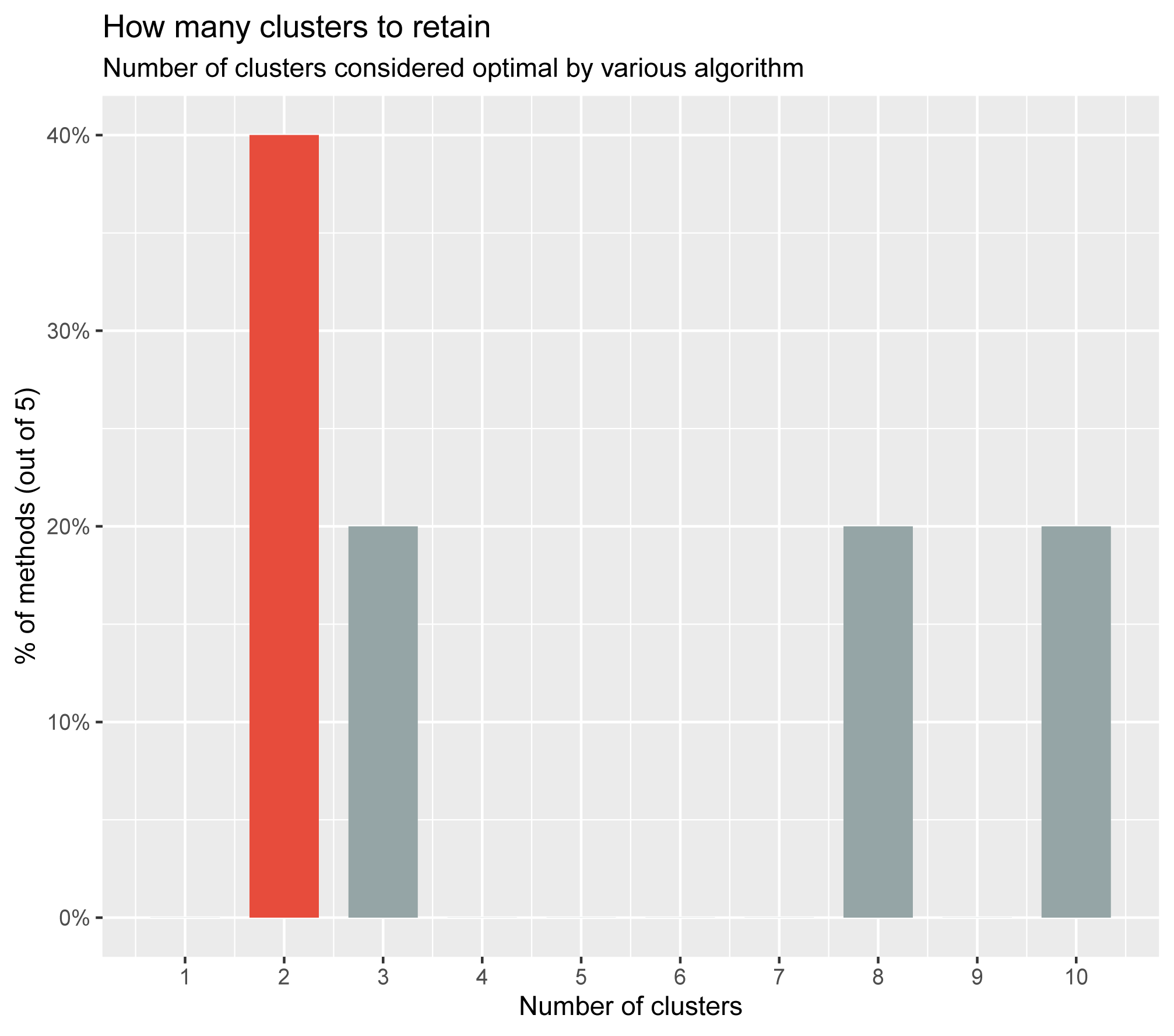


**Supplementary Figure 1.** **Determination of the optimal number of clusters using a consensus clustering strategy.**

**
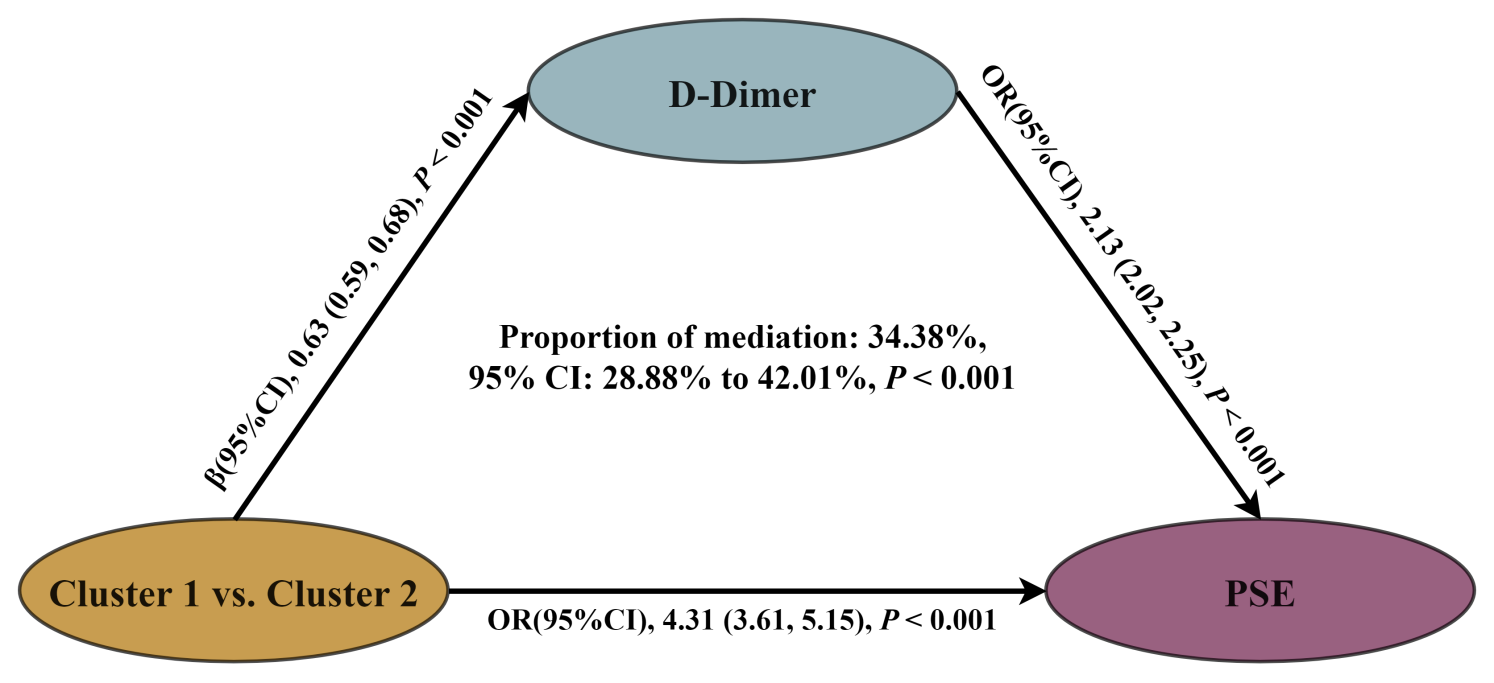
**

**Supplementary Figure 2. Mediation effects of D-dimer on the association between Cluster and PSE.** Adjusted for sex, diabetes, hypertension, coronary disease, atrial fibrillation, hydrocephalus, cortical involvement, large vessel disease, eGFR, alanine aminotransferase, urea, blood uric acid, platelet count, red blood cell count, and APTT. Cluster 1, inflammatory-neurological deficit cluster; Cluster 2, metabolic dysregulation cluster; OR, odds ratio; CI, confidence interval; eGFR, estimated glomerular filtration rate; APTT, activated partial thromboplastin time; PSE, post-stroke epilepsy.

**
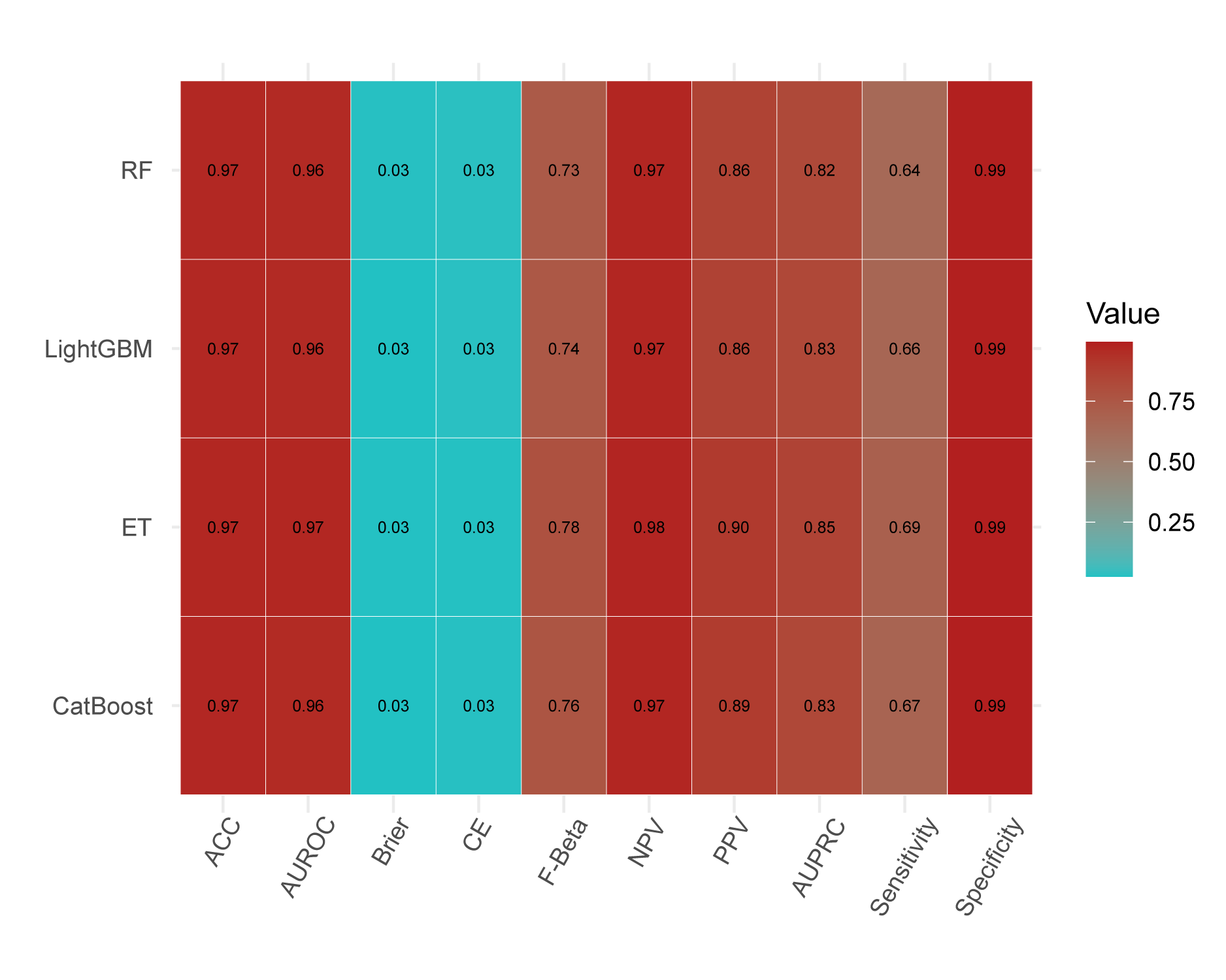
**

**Supplementary Figure 3. Performance heatmap comparing machine learning models of Benchmarking on the training sets for the inflammatory-neurological deficit cluster.** ET, Extra Trees; LightGBM, Light Gradient Boosting Machine; RF, Random Forest; ACC, Accuracy; AUROC, area under the receiver operating characteristic curve; Brier, Brier Score; CE, Cross Entropy; F-Beta, F-Beta Score; NPV, Negative Predictive Value; PPV, Positive Predictive Value; AUPRC, area under the precision-recall curve.

**
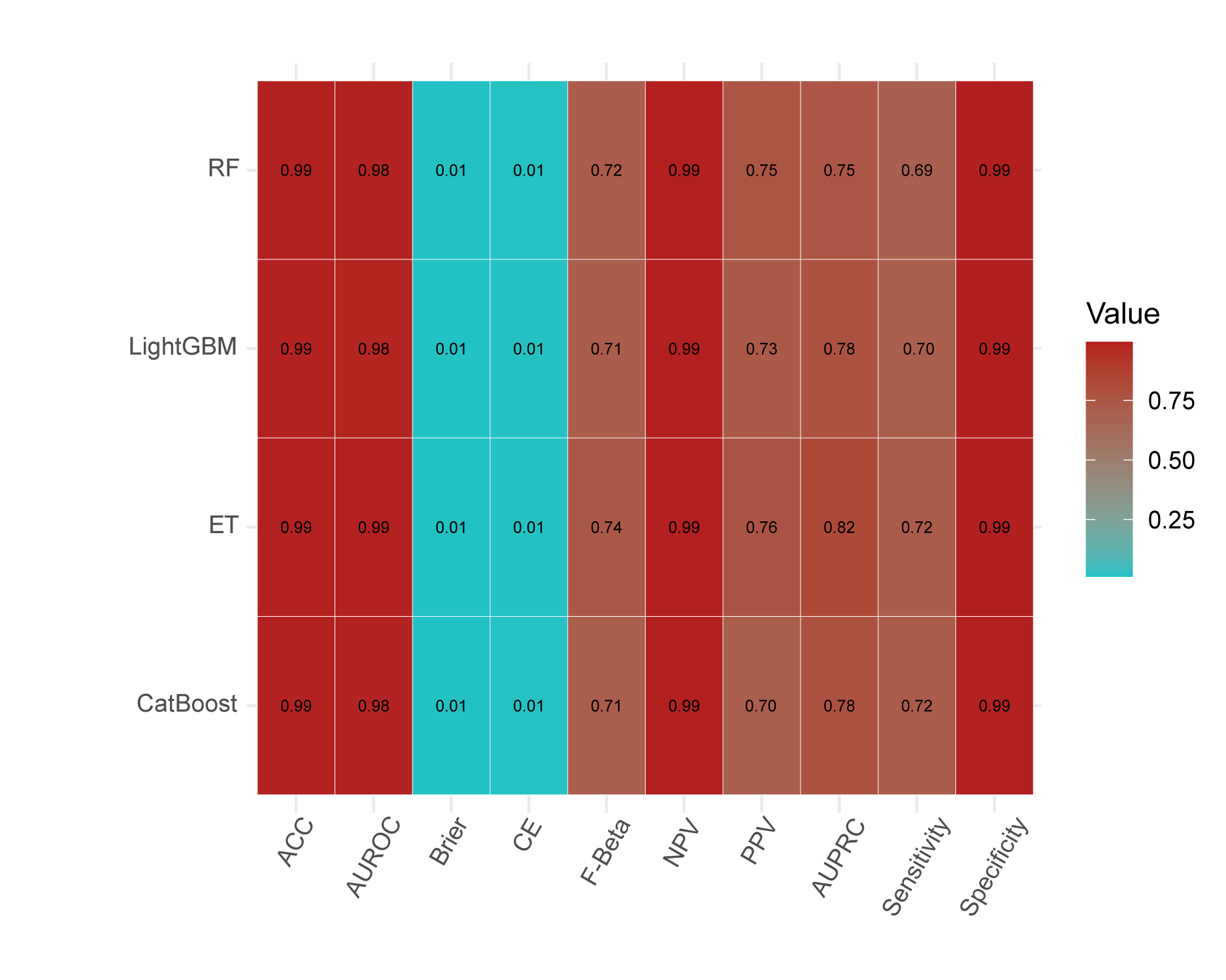
**

**Supplementary Figure 4. Performance heatmap comparing machine learning models of Benchmarking on the training sets for the metabolic dysregulation cluster.** ET, Extra Trees; LightGBM, Light Gradient Boosting Machine; RF, Random Forest; ACC, Accuracy; AUROC, area under the receiver operating characteristic curve; Brier, Brier Score; CE, Cross Entropy; F-Beta, F-Beta Score; NPV, Negative Predictive Value; PPV, Positive Predictive Value; AUPRC, area under the precision-recall curve.

**

**

**Supplementary Figure 5. Performance of the Extra Trees model of Benchmarking on the training sets for the inflammatory-neurological deficit cluster.** Receiver operating characteristic curve (**A**); Confusion matrix (**B**); Precision-recall curve (**C**); Calibration curve (**D**); Decision curve analysis (**E**); Metric radar plot (**F**). AUROC, area under the receiver operating characteristic curve; AUPRC, area under the precision-recall curve.





**Supplementary Figure 6. Performance of the Extra Trees model on the test set for the inflammatory-neurological deficit cluster.** Receiver operating characteristic curve (**A**); Confusion matrix (**B**); Precision-recall curve (**C**); Calibration curve (**D**); Decision curve analysis (**E**); Metric radar plot (**F**). AUROC, area under the receiver operating characteristic curve; AUPRC, area under the precision-recall curve.

**

**

**Supplementary Figure 7. Performance of the Extra Trees model of Benchmarking on the training sets for the metabolic dysregulation cluster.** Receiver operating characteristic curve (**A**); Confusion matrix (**B**); Precision-recall curve (**C**); Calibration curve (**D**); Decision curve analysis (**E**); Metric radar plot (**F**). AUROC, area under the receiver operating characteristic curve; AUPRC, area under the precision-recall curve.

**

**

**Supplementary Figure 8. Performance of the Extra Trees model on the test set for the metabolic dysregulation cluster.** Receiver operating characteristic curve (**A**); Confusion matrix (**B**); Precision-recall curve (**C**); Calibration curve (**D**); Decision curve analysis (**E**); Metric radar plot (**F**). AUROC, area under the receiver operating characteristic curve; AUPRC, area under the precision-recall curve.


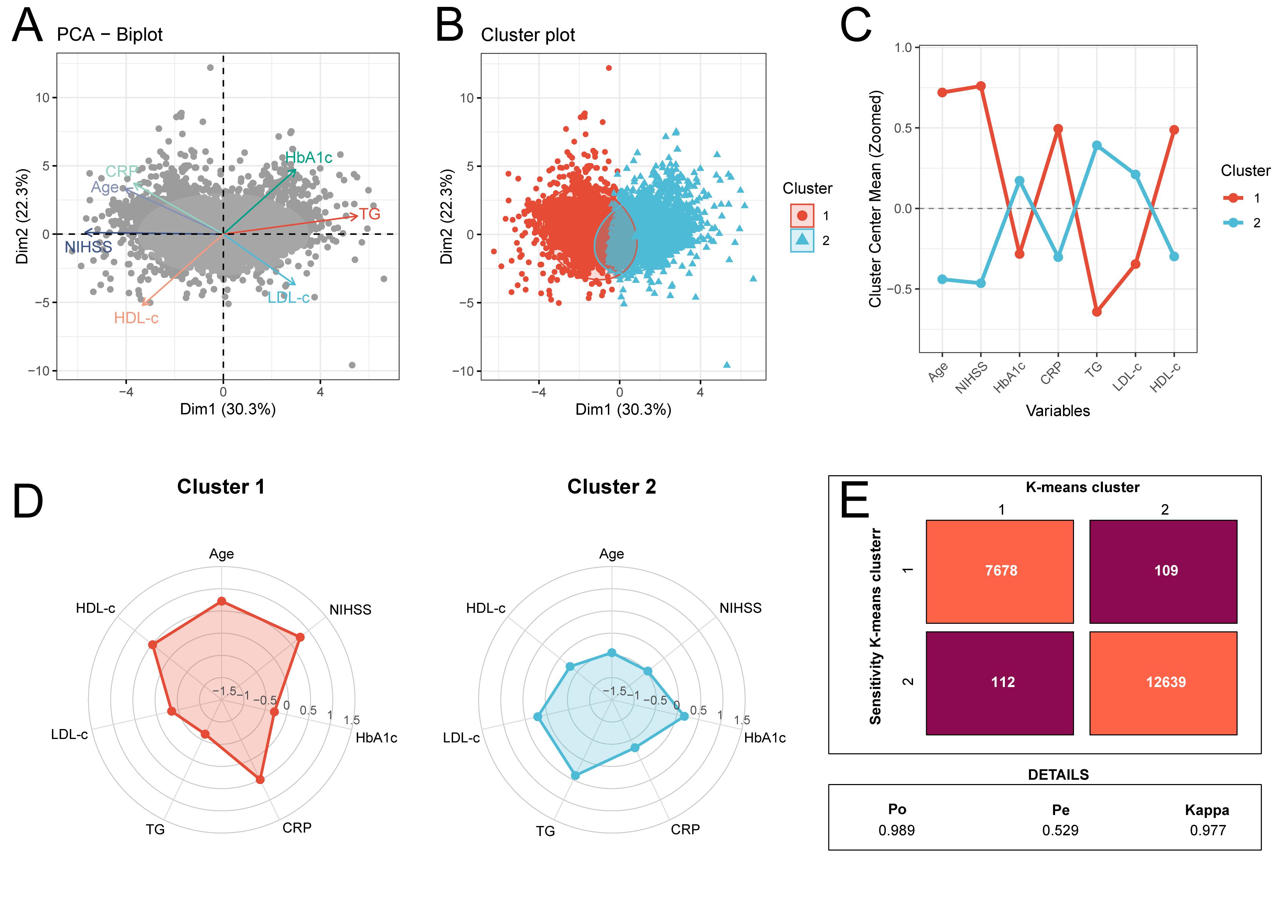


**Supplementary Figure 9. Identification of subtypes in middle-aged and older patients with AIS after winsorizing NIHSS, CRP, and TG at the 99th percentile.** PCA biplot (**A**); K-means clustering results (k = 2) (**B**); Cluster profiles (**C, D**); Confusion matrix (**E**). AIS, acute ischemic stroke; PCA, Principal Component Analysis; CRP, C-reactive protein; HDL-c, high-density lipoprotein cholesterol; NIHSS, National Institutes of Health Stroke Scale; HbA1c, glycated hemoglobin; LDL-c, low-density lipoprotein cholesterol; TG, triglycerides; PSE, post-stroke epilepsy; Po, Observed Agreement; Pe, Expected Agreement.


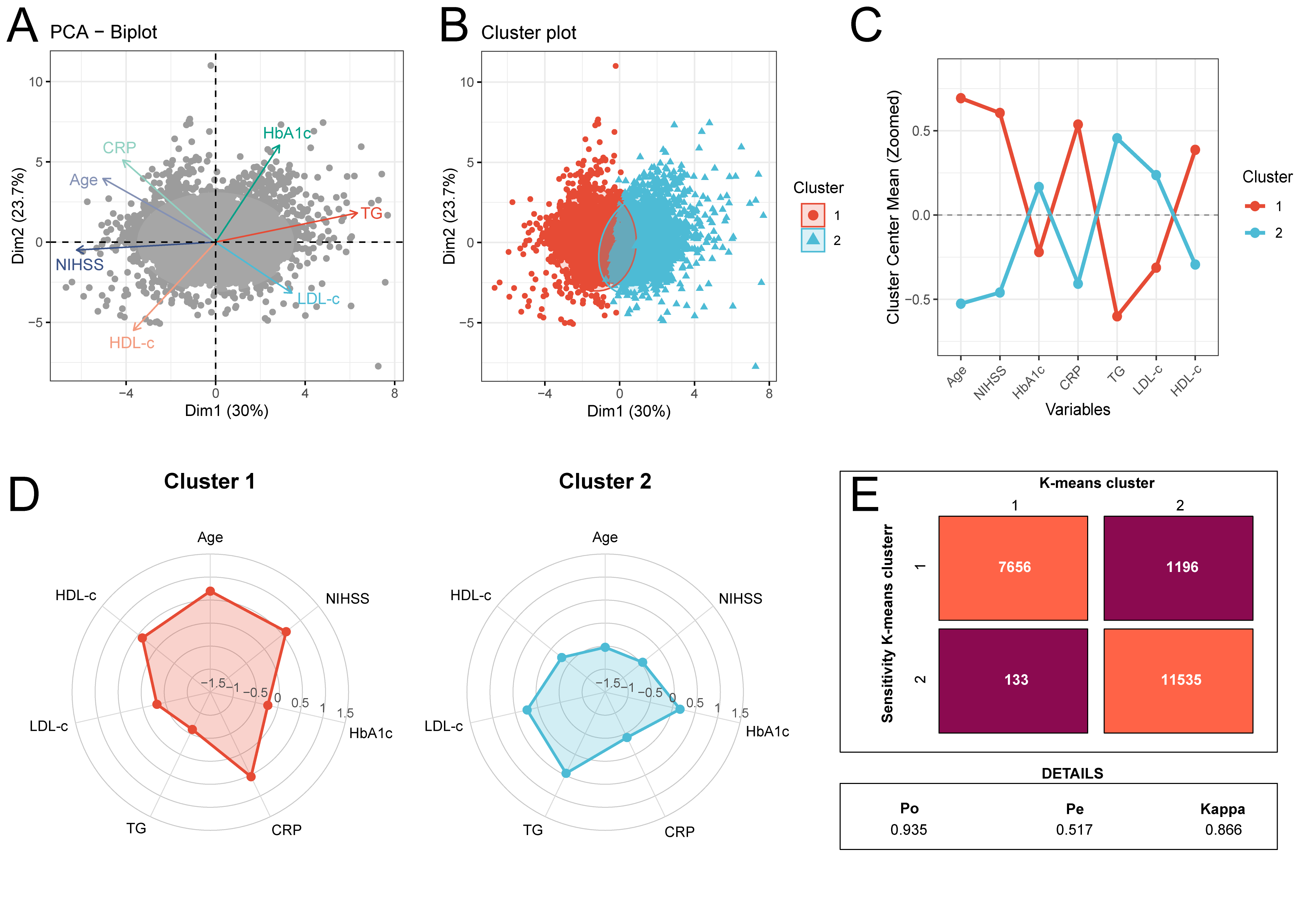


**Supplementary Figure 10. Identification of subtypes in middle-aged and older patients with AIS after excluding zero values (n = 18) and log-transforming NIHSS, CRP, and TG.** PCA biplot (**A**); K-means clustering results (k = 2) (**B**); Cluster profiles (**C, D**); Confusion matrix (**E**). AIS, acute ischemic stroke; PCA, Principal Component Analysis; CRP, C-reactive protein; HDL-c, high-density lipoprotein cholesterol; NIHSS, National Institutes of Health Stroke Scale; HbA1c, glycated hemoglobin; LDL-c, low-density lipoprotein cholesterol; TG, triglycerides; PSE, post-stroke epilepsy; Po, Observed Agreement; Pe, Expected Agreement.

# Supplementary Table

| **Supplementary Table 1.** Baseline characteristics of the study population according to PSE. | | | | |
| --- | --- | --- | --- | --- |
| **Variables** | **Total**  **(n = 20,538)** | **Non-PSE**  **(n = 19,671)** | **PSE**  **(n = 867)** | ***P*** |
|  |  |  |  |  |
| Age (years), Mean ± SD | 67.69 ± 10.93 | 67.71 ± 10.94 | 67.23 ± 10.84 | 0.205 |
| Sex, Female n (%) | 10,260 (49.96) | 9,737 (49.50) | 523 (60.32) | < 0.001 |
| NIHSS, Mean ± SD | 8.05 ± 2.96 | 7.90 ± 2.89 | 11.43 ± 2.55 | < 0.001 |
| Diabetes, n (%) | 7,167 (34.90) | 6,847 (34.81) | 320 (36.91) | 0.204 |
| Hypertension, n (%) | 14,308 (69.67) | 13,683 (69.56) | 625 (72.09) | 0.113 |
| Coronary disease, n (%) | 9,521 (46.36) | 9,180 (46.67) | 341 (39.33) | < 0.001 |
| Atrial fibrillation, n (%) | 2,024 ( 9.85) | 1,911 (9.71) | 113 (13.03) | 0.001 |
| Hydrocephalus, n (%) | 250 ( 1.22) | 206 (1.05) | 44 (5.07) | < 0.001 |
| Cortical involvement, n (%) | 1,255 ( 6.11) | 1,150 (5.85) | 105 (12.11) | < 0.001 |
| Large vessel disease, n (%) | 5,401 (26.30) | 5,175 (26.31) | 226 (26.07) | 0.875 |
| TG (mmol/L), Mean ± SD | 1.55 ± 0.43 | 1.55 ± 0.43 | 1.66 ± 0.49 | < 0.001 |
| LDL-c (mmol/L), Mean ± SD | 2.70 ± 0.36 | 2.70 ± 0.36 | 2.69 ± 0.38 | 0.318 |
| HDL-c (mmol/L), Mean ± SD | 1.25 ± 0.15 | 1.25 ± 0.15 | 1.24 ± 0.15 | 0.120 |
| HbA1c (%), Mean ± SD | 6.69 ± 0.93 | 6.69 ± 0.92 | 6.75 ± 0.99 | 0.062 |
| CRP (mg/L), Median (IQR) | 9.50 (4.80, 19.30) | 9.00 (4.70, 17.80) | 33.00 (20.85, 85.55) | < 0.001 |
| eGFR (mL/min/1.73m²), Mean ± SD | 88.82 ± 29.20 | 89.09 ± 29.41 | 82.66 ± 23.11 | < 0.001 |
| Alanine aminotransferase (U/L), Mean ± SD | 24.28 ± 10.29 | 24.16 ± 10.25 | 27.05 ± 10.74 | < 0.001 |
| Urea (mmol/L), Mean ± SD | 6.47 ± 1.41 | 6.48 ± 1.41 | 6.36 ± 1.30 | 0.019 |
| Blood uric acid (µmol/L), Mean ± SD | 343.15 ± 58.62 | 343.33 ± 57.92 | 339.04 ± 72.64 | 0.035 |
| Platelet count (10^9^/L), Mean ± SD | 189.62 ± 26.86 | 190.13 ± 26.36 | 177.86 ± 34.42 | < 0.001 |
| Red blood cell count (10^12^/L), Mean ± SD | 4.31 ± 0.32 | 4.30 ± 0.32 | 4.40 ± 0.26 | < 0.001 |
| APTT (s), Mean ± SD | 35.67 ± 2.32 | 35.69 ± 2.34 | 35.15 ± 1.88 | < 0.001 |
| D-dimer (µg/mL), Median (IQR) | 0.91 (0.65, 1.50) | 0.89 (0.64, 1.43) | 1.96 (1.38, 6.88) | < 0.001 |
| PSE, post-stroke epilepsy; NIHSS, National Institutes of Health Stroke Scale; TG, triglycerides; SD, standard deviation; IQR, interquartile range; LDL-c, low-density lipoprotein cholesterol; HDL-c, high-density lipoprotein cholesterol; HbA1c, glycated hemoglobin; CRP, C-reactive protein; eGFR, estimated glomerular filtration rate; APTT, activated partial thromboplastin time. | | | | |

| **Supplementary Table 2.** Hyperparameters of the Extra Trees model. | |
| --- | --- |
| **Hyperparameters** | **Value** |
| classif.extratrees.ntree | 500 |
| classif.extratrees.nodesize | 1 |
| classif.extratrees.numRandomCuts | 1 |
| classif.extratrees.evenCuts | FALSE |
| classif.extratrees.numThreads | 1 |
| classif.extratrees.numRandomTaskCuts | 1 |

| **Supplementary Table 3.** Associations between Cluster and PSE after winsorizing NIHSS, CRP, and TG at the 99th percentile. | | | | | | | | |
| --- | --- | --- | --- | --- | --- | --- | --- | --- |
| **Variable** | **Crude** | | **Model 1** | | **Model 2** | | **Model 3** | |
|  | **OR (95%CI)** | ***P*** | **OR (95%CI)** | ***P*** | **OR (95%CI)** | ***P*** | **OR (95%CI)** | ***P*** |
| **Metabolic dysregulation cluster** | 1(Ref) |  | 1(Ref) |  | 1(Ref) |  | 1(Ref) |  |
| **Inflammatory-neurological deficit cluster** | 3.14 (2.72~3.62) | <0.001 | 3.74 (3.21~4.36) | <0.001 | 3.67 (3.14~4.28) | <0.001 | 4.29 (3.59~5.12) | <0.001 |
| Model 1: Adjusted for sex, diabetes, hypertension, coronary disease, atrial fibrillation, and hydrocephalus. Model 2: Adjusted for sex, diabetes, hypertension, coronary disease, atrial fibrillation, hydrocephalus, cortical involvement, and large vessel disease. Model 3: Adjusted for sex, diabetes, hypertension, coronary disease, atrial fibrillation, hydrocephalus, cortical involvement, large vessel disease, eGFR, alanine aminotransferase, urea, blood uric acid, platelet count, red blood cell count, and APTT. OR, odds ratio; CI, confidence interval; eGFR, estimated glomerular filtration rate; APTT, activated partial thromboplastin time; PSE, post-stroke epilepsy. | | | | | | | | |

| **Supplementary Table 4.** Associations between Cluster and PSE after excluding zero values (n = 18) and log-transforming NIHSS, CRP, and TG. | | | | | | | | |
| --- | --- | --- | --- | --- | --- | --- | --- | --- |
| **Variable** | **Crude** | | **Model 1** | | **Model 2** | | **Model 3** | |
|  | **OR (95%CI)** | ***P*** | **OR (95%CI)** | ***P*** | **OR (95%CI)** | ***P*** | **OR (95%CI)** | ***P*** |
| **Metabolic dysregulation cluster** | 1(Ref) |  | 1(Ref) |  | 1(Ref) |  | 1(Ref) |  |
| **Inflammatory-neurological deficit cluster** | 3.16 (2.72~3.66) | <0.001 | 3.57 (3.05~4.18) | <0.001 | 3.51 (3.00~4.11) | <0.001 | 4.23 (3.53~5.07) | <0.001 |
| Model 1: Adjusted for sex, diabetes, hypertension, coronary disease, atrial fibrillation, and hydrocephalus. Model 2: Adjusted for sex, diabetes, hypertension, coronary disease, atrial fibrillation, hydrocephalus, cortical involvement, and large vessel disease. Model 3: Adjusted for sex, diabetes, hypertension, coronary disease, atrial fibrillation, hydrocephalus, cortical involvement, large vessel disease, eGFR, alanine aminotransferase, urea, blood uric acid, platelet count, red blood cell count, and APTT. OR, odds ratio; CI, confidence interval; eGFR, estimated glomerular filtration rate; APTT, activated partial thromboplastin time; PSE, post-stroke epilepsy. | | | | | | | | |
